# Intermittent Hypoxia-Induced Activation of Endothelial Cells Is Mediated *via* Sympathetic Activation-Dependent Catecholamine Release

**DOI:** 10.3389/fphys.2021.701995

**Published:** 2021-07-12

**Authors:** Rengul Cetin-Atalay, Angelo Y. Meliton, David Wu, Parker S. Woods, Kaitlyn A. Sun, Ying-Jie Peng, Jayasri Nanduri, Xiaoyu Su, Yun Fang, Robert B. Hamanaka, Nanduri Prabhakar, Gökhan M. Mutlu

**Affiliations:** ^1^Department of Medicine, University of Chicago, Chicago, IL, United States; ^2^Section of Pulmonary and Critical Care Medicine, University of Chicago, Chicago, IL, United States; ^3^Section of Emergency Medicine, University of Chicago, Chicago, IL, United States; ^4^Institute for Integrative Physiology, University of Chicago, Chicago, IL, United States

**Keywords:** obstructive sleep apnea, endothelial cell, epinephrine (adrenaline), intermittent hypoxia, inflammation

## Abstract

Obstructive sleep apnea (OSA) is a common breathing disorder affecting a significant percentage of the adult population. OSA is an independent risk factor for cardiovascular disease (CVD); however, the underlying mechanisms are not completely understood. Since the severity of hypoxia correlates with some of the cardiovascular effects, intermittent hypoxia (IH) is thought to be one of the mechanisms by which OSA may cause CVD. Here, we investigated the effect of IH on endothelial cell (EC) activation, characterized by the expression of inflammatory genes, that is known to play an important role in the pathogenesis of CVD. Exposure of C57BL/6 mice to IH led to aortic EC activation, while *in vitro* exposure of ECs to IH failed to do so, suggesting that IH does not induce EC activation directly, but indirectly. One of the consequences of IH is activation of the sympathetic nervous system and catecholamine release. We found that exposure of mice to IH caused elevation of circulating levels of catecholamines. Inhibition of the IH-induced increase in catecholamines by pharmacologic inhibition or by adrenalectomy or carotid body ablation prevented the IH-induced EC activation in mice. Supporting a key role for catecholamines, epinephrine alone was sufficient to cause EC activation *in vivo* and *in vitro*. Together, these results suggested that IH does not directly induce EC activation, but does so indirectly *via* release of catecholamines. These results suggest that targeting IH-induced sympathetic nerve activity and catecholamine release may be a potential therapeutic target to attenuate the CV effects of OSA.

## Introduction

Obstructive sleep apnea (OSA) is a common breathing disorder affecting a significant percentage of the adult population. When defined as an apnea-hypopnea index of 5 or greater, the prevalence of OSA was estimated to be 24% in men and 9% in women aged 30–60 years (Young et al., 1993). According to population-based studies, the prevalence of OSA with daytime sleepiness is approximately 3–7% in men and 2–5% in women (Punjabi, 2008). A recent literature-based analysis of global prevalence estimated that 936 million adults between the ages of 30 and 69 have mild to severe OSA and 425 million adults have moderate to severe OSA (Benjafield et al., 2019). Patients with OSA have a high prevalence of comorbidities, including hypertension and cardiovascular disease (CVD; Robichaud-Halle et al., 2012; Appleton et al., 2018; Ruel et al., 2018; Tveit et al., 2018). Indeed, OSA is an independent risk factor for CVD (Young et al., 1993, 2009; Somers et al., 1995; Fletcher, 2001; Neubauer, 2001; Marin et al., 2005; Yaggi et al., 2005; Marshall et al., 2008; Punjabi, 2008; Dempsey et al., 2010; AASM, 2014). The frequency of CVD increases with the severity of OSA (Marin et al., 2005), and some of the cardiovascular effects of OSA, such as hypertension, show linear correlation with the severity of hypoxia (McNicholas et al., 2007). However, the underlying molecular mechanisms by which OSA causes hypertension and CVD are not completely understood.

Mechanistically, OSA is characterized by collapse of the upper airway causing recurrent pauses in breathing during sleep leading to intermittent hypoxemia (IH), arousals, changes in intrathoracic pressure, and sleep fragmentation (Somers et al., 1995; Gabryelska et al., 2018). Furthermore, patients with OSA exhibit increased sympathetic nerve activity (Dempsey et al., 2010; Prabhakar, 2016) as well as elevated circulating and urinary catecholamines (both norepinephrine and epinephrine), which are attributed to heightened sympathetic nerve activity (Fletcher et al., 1987; Carlson et al., 1993; Marrone et al., 1993; Somers et al., 1995; Peng et al., 2021). IH is a hallmark manifestation of OSA and thought to be a major factor contributing to the pathogenesis of OSA-related cardiovascular morbidity. However, the specific roles that IH and heightened sympathetic nerve activity play in the pathogenesis of cardiovascular morbidity seen in OSA are not completely understood.

Endothelial cell (EC) activation is an early pathophysiologic event in the development of CVD. We have recently shown that EC activation is linked to the flow pattern in vasculature and occurs at where blood flow is disturbed, such as arterial bifurcation sites (i.e., aortic arch), where atherosclerosis is more likely to develop (Wu et al., 2017). When activated, ECs become pro-inflammatory due to increased expression of cytokines and cell adhesion molecules (Hunt and Jurd, 1998). EC activation then leads to leukocyte adhesion, macrophage activation, and platelet aggregation, all of which contribute to risk of developing CVD (Dyugovskaya et al., 2002; Arnaud et al., 2011; Hopkins, 2013). EC activation may play a key role in linking OSA to CVD (Hunt and Jurd, 1998; Deanfield et al., 2007). Humans with OSA exhibit markers of EC activation, such as elevated levels of pro-inflammatory cytokines, and cell adhesion molecules, such as vascular cell adhesion molecule 1 and intercellular adhesion molecule 1 (Hunt and Jurd, 1998; Dyugovskaya et al., 2002; Jelic et al., 2008; Arnaud et al., 2011). Importantly, treatment of OSA with continuous positive airway pressure (CPAP) reverses the effects of OSA on markers of EC activation (Nadeem et al., 2013; Xie et al., 2013). However, patient compliance with CPAP is low due to mask- or pressure-related discomfort and long-term adherence to CPAP therapy remains unacceptable (<50%). Thus, there is unmet need to better understand the mechanisms by which OSA affects the cardiovascular system and induces EC activation so that therapies other than CPAP, including pharmacologic treatments, may be initiated in those individuals who fail to respond to or are not compliant with CPAP therapy.

Here, we demonstrate how OSA causes EC activation using *in vivo* and *in vitro* models of IH. We found that while *in vivo* exposure to IH induced EC activation in aortic ECs from mice, it failed to do so in cell cultures of ECs *in vitro*. Because IH increases sympathetic nerve activity, we sought to investigate whether IH induces EC activation through increased catecholamine levels resulting from activation of the sympathetic nervous system. We confirmed that IH increases systemic catecholamine levels. Pharmacologic inhibition of catecholamine release with the treatment of 6-hydroxydopamine, a sympatholytic agent, prevented IH-induced EC activation in mice. Furthermore, inhibition of IH-induced catecholamine release by adrenalectomy or carotid body ablation also prevented IH-induced EC activation. Consistent with a key role for catecholamines in EC activation, we found that epinephrine alone was sufficient to induce EC activation *in vitro* and *in vivo*. We concluded that IH causes EC activation not directly, but indirectly *via* epinephrine, which is released due to IH-induced heightened sympathetic nerve activity. Our findings suggest that targeting IH-induced sympathetic nerve activity and catecholamine release may be a potential therapeutic approach to attenuate the CV effects of OSA.

## Materials and Methods

### Animals

All animal experiments and procedures were performed according to the protocols approved by the Institutional Animal Care and Use Committee at the University of Chicago. We dissected aorta from 6- to 8-week-old C57BL/6 mice and bilateral adrenalectomized mice (Jackson Laboratory) as previously described (Chiarella et al., 2014; Wu et al., 2017). We then isolated RNA from aortic endothelial cells isolated by gentle and slow flushing with TRI Reagent (Sigma). Prior to dissection, blood samples for catecholamine levels were collected from right ventricle and 5 ml of PBS was injected into the left ventricle in order to flush blood out of the aortic lumen.

#### Intermittent Hypoxia

For *in vivo* exposure to IH, mice were placed in a specialized chamber, which is flushed with cycles of pure nitrogen and compressed air following a chronic IH protocol consisting of 15 s of 5% O_2_ followed by 5 min of 21% O_2_ (normoxia), 9 episodes/h and 8 h per day for 10 days as previously described (Peng et al., 2006).

#### Chemical Sympathectomy

Endogenously produced catecholamines were depleted by treating mice with 6-hydroxydopamine (6-OHDA; Sigma; 100 mg/kg in 100 μl solution) intraperitoneally on days 1, 3, and 8 as previously described (Kostrzewa and Jacobowitz, 1974).

#### Administration of Epinephrine

To determine the effects of elevated catecholamines, we placed Alzet pumps subcutaneously in the interscapular area of mice 3 days prior to epinephrine infusion. Pumps were loaded with epinephrine to deliver the drug at 5 mg/kg/24 h continuously for 10 days.

#### Adrenalectomy and Carotid Body Ablation

We purchased adrenalectomized mice directly from Jackson laboratory. We performed carotid body ablation as we have previously described (Kumar et al., 2015; Nanduri et al., 2018).

### *In vitro* Exposure to Intermittent Hypoxia

For *in vitro* studies, human aortic endothelial cells (HAECs) were purchased from Lonza (Allendale, NJ; CC-2535, lot number 000022494 or 000035034). Cells were grown in EGM-2 supplemented with SingleQuots from Lonza (CC-3156 and CC-4176) supplemented with Penicillin/Streptomycin/Amphotericin B Solution (Sigma). All primary cultures were used from passage 5 to 7. Cell cultures were exposed intermittent hypoxia in parallel to control (normoxia) as described previously (Yuan et al., 2005). Briefly, cells were treated with alternating cycles of 1.5% O_2_ for 30 s followed by 20% O_2_ for 5 min for 60 cycles in a temperature- and humidity-controlled chamber (Yuan et al., 2005). The CO_2_ level in the control normoxia-exposed cells and during IH exposure was maintained at 10%, and balance was N_2_ as we previously described (Yuan et al., 2005). Ambient O_2_ levels in the IH chamber were monitored by an O_2_ analyzer (Alpha Omega Instruments, Huston, TX). We did not observe any morphological changes in ECs following IH exposure (Supplementary Figure S1).

### RNA Isolation and Gene Expression

Total RNA was isolated from cells using TRI Reagent (Zymo Research, R2050-1-200) and Zymo Direct-zol RNA Miniprep Kit (Zymo Research, catalog number R2053) and reverse-transcribed using Bio-Rad iScript Reverse Transcription Supermix (Bio-Rad, catalog #1708841) in a Bio-Rad C1000 Touch Thermal Cycler as previously described (Sun et al., 2020). Quantitative mRNA expression was determined by real-time qPCR using iTaq Universal SYBR Green Supermix (Bio-Rad, catalog #172-5121). Rpl19 (Ribosomal protein L19) used as a housekeeping gene, and gene expression was quantified using the ΔΔCt method to determine the relative fold change. The human- and mouse-specific primer sequences used for quantitative-PCR are as follows:

The following mouse-specific primer sequences were used:

*rpl19* 5'-CCGACGAAAGGGTATGCTCA-3', 5'-GACCTTCTTTTTCCCGCAGC-3',

*il6* 5'-GAGGATACCACTCCCAACAGACC -3', 5'-AAGTGCATCATCGTTGTTCATACA - 3'

*kc* 5'-AGACCATGGCTGGGATTCAC-3', 5'-ATGGTGGCTATGACTTCGGT-3'

*vcam1* 5'- TATGTCAACGTTGCCCCCAA-3', 5'- ATGCCGGAATCGTCCCTTTT-3'

*icam1* 5'- GCCTCCGGACTTTCGATCTT-3', 5'-CTGGTCCGCTAGCTCCAAAA-3'.

The following human-specific primer sequences were used:

*RPL19* 5'-AGTATGCTCAGGCTTGAGAAGA-3', 5'-CATTGGTCTCATTGGGGTCTAAC-3'.

*IL6* 5'-GCCCTGAGAAAGGAGACATGTAA-3', 5'-TTGTTTTCTGCCAGTGCCTC-3'.

*IL8* 5'-AGAGAGCTCTGTCTGGACCC-3', 5'-TCTCAGCCCTCTTCAAAAACTTC-3'.

*VCAM1* 5'-GATACAACCGTCTTGGTCAG-3' 5'-TAATTCCTTCACATAAATAAACCC-3'.

*ICAM1* 5'-ACGGAGCTCCCAGTCCTAAT-3' 5'-CTCCTTCTGGGGAAAGGCAG'-3'.

### Leukocyte Adhesion Assay

Leukocyte adhesion studies were done as we previously described (Wu et al., 2017). We used THP-1 cells, a human monocyte cell line, which were grown in suspension in RPMI media (Thermo Fisher, Waltham, MA) supplemented with 10% FBS (Biowest USA, Riverside, MO). We first treated HAECs with epinephrine (10 μm) or control for 24 h. For adhesion studies, the cells were incubated with 5 μm Calcein acetoxymethyl (AM) dye (Thermo Fisher) for 30 min at 37°C and then resuspended in 600 μl serum-free RPMI medium (900 rpm for 5 min at room temperature). HAECs were washed twice, and the medium was replaced with regular EGM2 medium (1 ml). THP-1 cells were then added on to the HAECs and incubated for 1 h at 37°C; cells were rocked gently twice after 30 min. The HAECs+THP-1 cells were then washed gently with warm PBS five times. Fluorescence of the Calcein AM dye was then quantified in area scanning mode of Cytation 3 (Biotek, Winooski, VT) device, with gain of 80, and excitation 492 nm, and emission 550 nm.

### Statistics

The data were analyzed in Prism 8 (GraphPad Software Inc., La Jolla, CA). All data are shown as mean ± SEM. Significance was determined by unpaired, two-tailed Student’s *t*-test (for comparisons between two samples) or by one-way ANOVA using Bonferroni’s correction for multiple comparisons. Statistical significance was defined as ^*^p < 0.05, ^**^p < 0.005, ^***^p < 0.001, and ^****^p < 0.0001.

## Results

### Intermittent Hypoxia Induces Aortic EC Activation *in vivo* but Not *in vitro*

**Figure 1:**
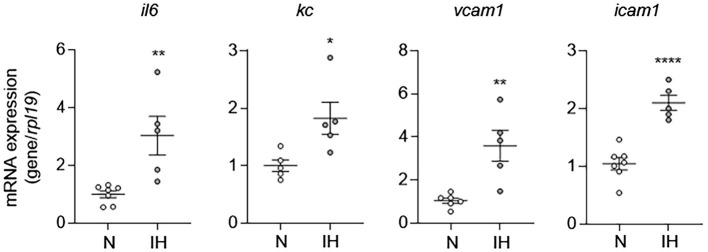
Intermittent hypoxia induces activation of aortic endothelial cells (ECs) in mice. We exposed C57BL/6 mice to either normoxia (N) or IH for 10 days and removed and flushed aorta with TRI Reagent to isolate RNA from aortic ECs and then assessed EC activation by measuring mRNA expression of markers of EC activation genes, including *il6*, *kc*, *vcam1*, and *icam1. N* = 5–6. Significance was determined by Student’s *t*-test. ^*^p < 0.05, ^**^p < 0.005, and ^****^p < 0.0001.

Given that EC activation plays an important role in the pathogenesis of CVD, we first sought to determine whether IH causes EC activation as a mechanism to explain the increased prevalence of CVD in OSA. To do this, we exposed C57BL/6 mice to either IH or normoxia (room air) for 10 days. At the end of experiments, we removed the whole aorta from mice and then extracted RNA from isolated ECs as we previously described (Wu et al., 2017) and performed qPCR for markers of EC activation (*il6*, *kc*, *vcam1*, and *icam1*). Compared to aortic ECs from normoxia-exposed control mice, aortic ECs from mice exposed to IH exhibited higher expression of markers of EC activation consistent with the finding that IH causes EC activation in mice (Figure 1).

**Figure 2:**
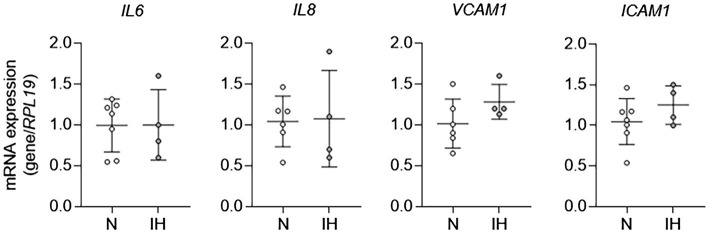
Intermittent hypoxia alone fails to induce aortic EC activation *in vitro*. We exposed human aortic ECs to either normoxia (N) or IH for 60 cycles and then assessed EC activation by measuring mRNA expression of markers of EC activation genes, including *IL6*, *IL8, VCAM1*, and *ICAM1. N* = 4–6.

To study the mechanisms by which IH causes EC activation in mice further, we exposed HAECs to 60 cycles of IH *in vitro* and performed qPCR for markers of EC activation. In contrast to *in vivo* exposure to IH, mRNA expression of markers of EC activation [*IL6*, *IL8* (human homologue of *kc*), *VCAM1*, and *ICAM1*] was not different between control ECs and those exposed to IH *in vitro* (Figure 2). Collectively, these results show that while IH induces EC activation *in vivo*, it does not have an effect on EC activation *in vitro*. These results suggested that EC activation by IH may be mediated by an indirect effector that is regulated by IH exposure.

### Intermittent Hypoxia Increases Systemic Catecholamine Levels in Mice

**Figure 3:**
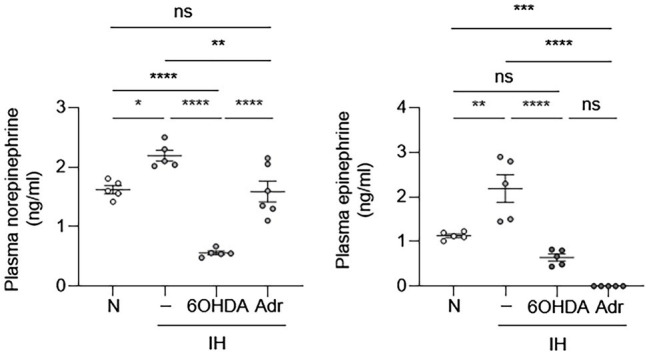
Intermittent hypoxia increases systemic catecholamine levels. We exposed C57BL/6 mice to either normoxia (N) or IH for 10 days in the absence or presence of 6-hydroxydopamine (6OHDA), which depletes catecholamines. We also exposed adrenalectomized (Adr) mice to IH for 10 days. At the end of experiments, we collected plasma and measured catecholamine (epinephrine and norepinephrine) levels. *N* = 5–6. Significance was determined by one-way ANOVA using Bonferroni’s correction. ns, not significant. ^*^p < 0.05, ^**^p < 0.005, ^***^p < 0.001, and ^****^p < 0.0001.

Our results suggested that EC activation may be an indirect effect of IH. One of the clinical consequences of IH is sympathetic nervous system activation, which results in catecholamine release (Kumar et al., 2006). Therefore, we first sought to determine whether IH can increase sympathetic nerve activity and cause increased release of catecholamines in mice. We exposed mice to IH for 10 days and then obtained blood samples to measure epinephrine and norepinephrine levels. Compared to the control group, mice exposed to IH had higher systemic levels of catecholamines (Figure 3). This increase in plasma catecholamine levels was abolished in the presence of 6-hydroxydopamine, which causes chemical sympathectomy and in adrenalectomized mice (Figure 3). These results are consistent with our previously published work, which showed that carotid body ablation inhibits IH-induced increase in systemic levels of catecholamines (Peng et al., 2014).

### Intermittent Hypoxia-Induced EC Activation in Mice Requires Catecholamines

**Figure 4:**
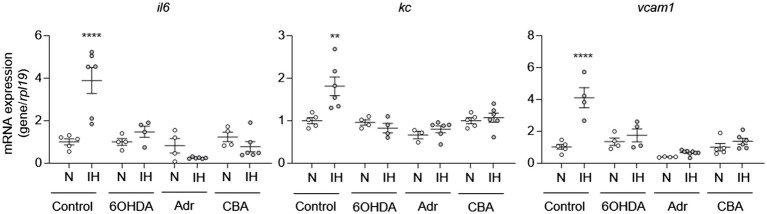
Intermittent hypoxia-induced catecholamines are required for intermittent hypoxia-induced aortic EC activation in mice. We exposed C57BL/6 mice to normoxia (N) or IH for 10 days in the absence or presence of 6OHDA. We also exposed mice with adrenalectomy (Adr) and carotid body ablation (CBA) to IH for 10 days. We then assessed EC activation by measuring mRNA expression of markers of EC activation genes, including *il6*, *kc*, and *vcam1. N* = 5–6. Significance was determined by one-way ANOVA using Bonferroni’s correction. ^**^p < 0.005; ^****^p < 0.0001.

To determine whether IH-induced elevation of systemic catecholamines may play a role in IH-induced aortic EC activation, we exposed mice to IH and treated them with 6-hydroxydopamine or vehicle. At the end of exposure, we removed the aortas to measure mRNA expression of *il6*, *kc*, and *vcam1* from ECs. Compared to vehicle treatment, 6-hydroxydopamine inhibited the IH-induced increase in the mRNA expression of *il6*, *kc*, and *vcam1* (Figure 4).

IH activates the sympathetic nervous system through carotid body chemoreflex [see review (Prabhakar, 2016)]. The adrenal medulla is a major end organ of the sympathetic nervous system and either adrenalectomized rats (Lesske et al., 1997) or surgical ablation of sympathetic nerves innervating adrenal medulla or the carotid bodies prevent elevated circulating catecholamine levels and hypertension in IH-treated rodents (Peng et al., 2014; Kumar et al., 2015). To further evaluate the role of catecholamines in IH-induced EC activation, we exposed mice with either adrenalectomy or carotid body ablation to IH for 10 days and evaluated EC activation. As expected, these mice did not exhibit upregulation of circulating catecholamines in response to IH (Figure 3; Peng et al., 2014). Compared to control mice, mice with adrenalectomy or carotid body ablation did not exhibit aortic EC activation in response to IH (Figure 4). Collectively, these results suggested that IH-induced EC activation *in vivo* is mediated *via* sympathetic nervous system activation and requires catecholamines.

### Epinephrine Alone Is Sufficient to Cause EC Activation *in vitro and in vivo*

**Figure 5:**
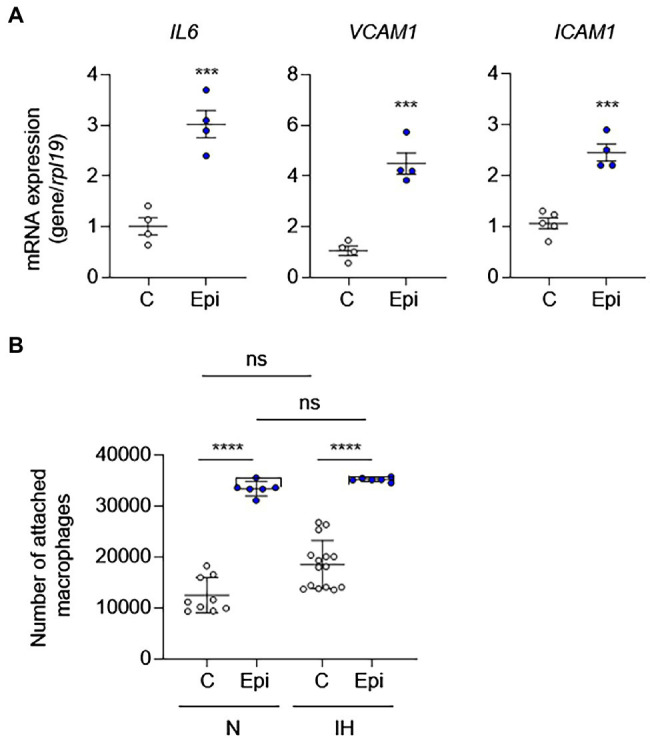
Epinephrine alone is sufficient to induce EC activation and leukocyte adhesion in the absence of IH *in vitro*. We treated ECs with epinephrine (Epi; 10 μm) or vehicle control (C) under normoxia for 24 h and then **(A)** assessed EC activation by measuring mRNA expression of markers of EC activation genes, including *IL6*, *VCAM1*, and *ICAM1* (*N* = 4; significance was determined by Student’s *t*-test) and **(B)** leukocyte adhesion (*N* = 8–10; significance was determined by one-way ANOVA using Bonferroni’s correction for multiple comparisons). ns, not significant. ^***^p < 0.001; ^****^p < 0.0001.

To determine whether catecholamines are responsible for IH-induced EC activation, we treated HAECs with epinephrine or norepinephrine as well as phenylephrine (an α-adrenergic receptor agonist) to determine whether direct stimulation with catecholamines is sufficient to induce EC activation. In contrast to IH exposure, epinephrine was sufficient to induce the mRNA expression EC activation markers, suggesting that increased levels of catecholamines may be responsible for the IH-induced EC activation (Figure 5A). In contrast to epinephrine, norepinephrine or phenylephrine failed to induce EC activation markers (Supplementary Figure S2). As leukocyte adhesion is associated with EC activation, we also measured leukocyte adhesion in HAECs exposed to IH or normoxia in the absence or presence of epinephrine. Consistent with the lack of EC activation after exposure to IH, IH did not significantly increase leukocyte adhesion to HAECs compared with normoxia-exposed cells. However, treatment with epinephrine increased leukocyte adhesion in both normoxic and IH-exposed cells (Figure 5B).

**Figure 6:**
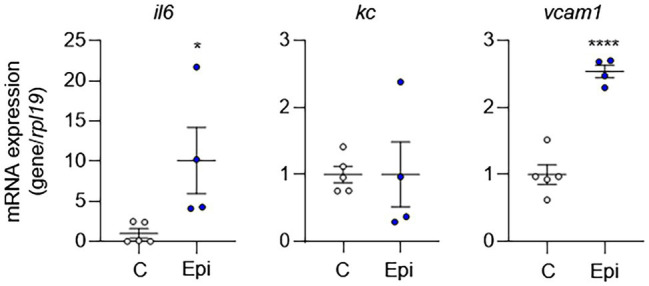
Epinephrine alone is sufficient to induce EC activation *in vivo*. We placed Alzet pumps in C57BL/6 mice to deliver epinephrine (Epi) or control vehicle (C) continuously for 10 days. We then assessed EC activation by measuring mRNA expression of markers of EC activation genes, including *il6*, *kc*, and *vcam1. N* = 4–5. Significance was determined by Student’s *t*-test. ^*^p < 0.05; ^****^p < 0.0001.

To complement our *in vitro* findings, we placed Alzet pumps to deliver epinephrine continuously to C57BL/6 mice for 10 days and then removed aortas to evaluate EC activation. Compared to control mice, epinephrine infusion increased the mRNA expression of EC activation markers (Figure 6). However, epinephrine infusion alone failed to increase *Kc* expression suggesting that other mechanisms may be involved. Together, these results suggest that epinephrine alone is sufficient to induce EC activation and leukocyte adhesion and further supports our finding that IH-induced EC activation mediated *via* sympathetic activation-derived catecholamines.

## Discussion

OSA is associated with significant morbidity and mortality due to CVD. In fact, OSA is independent risk factor for CVD; however, the mechanisms behind this correlation are not completely understood. While CPAP is effective in treating OSA, due to intolerance, compliance with CPAP remains unacceptably low. Therefore, there is unmet need to better understand how OSA affects cardiovascular system so that treatments alternative to CPAP can be developed.

In this study, we show that exposure of mice to IH induces EC activation, which plays a critical role in the pathogenesis of CVD. However, when we tried to study the effect of IH on EC activation *in vitro*, IH alone failed to induce EC activation. This lack of *in vitro* IH effect prompted us to explore other possibilities that may explain how IH induces EC activation *in vivo*. Since IH induces sympathetic nervous system activation (Kumar et al., 2006) and causes the release of catecholamines, we sought to determine how IH changes catecholamines *in vivo*. We found that exposure to IH increased plasma levels of both epinephrine and norepinephrine. Pharmacologic inhibition of IH-induced catecholamine release prevented the IH-induced EC activation in mice suggesting that it is the IH-induced sympathetic nervous system-derived catecholamines that are responsible for the EC activation. We confirmed these findings in mice with adrenalectomy and carotid body ablation.

We also confirmed the role of catecholamines in EC activation *in vitro*. Treatment of ECs with epinephrine alone was sufficient to induce EC activation as well as adhesion of macrophages. Neither norepinephrine nor phenylephrine induced EC activation suggesting that it is the engagement of β-adrenergic receptors that are required for EC activation. Catecholamines mediate their effect *via* α- and/or β-adrenergic receptors. ECs express both α- and β-adrenergic receptors (Sorriento et al., 2011); however, the relative expression of adrenergic receptor subtypes may differ depending on the vascular bed. Adrenergic receptors are G-protein-coupled receptors mediating their effects *via* second messengers, diacylglycerol and inositol 1,4,5-trisphosphate and cAMP, which affect numerous cell signaling pathways. Depending on the relative expression or density of adrenergic receptor subtypes, the effect of catecholamines on different EC populations may differ. For example, α_1_- and β_2_-adrenergic receptors mediate opposite effects on EC proliferation, neo-angiogenesis, and regulation of the vascular tone. While activation of α_1_-adrenergic receptor inhibits EC proliferation, neo-angiogenesis, and nitric oxide synthesis, β_2_-adrenergic receptor stimulates these processes (Sorriento et al., 2011). While our results suggest that β_2_-adrenergic receptors are important in the IH-induced EC activation, further studies will be needed to determine the precise role of catecholamines and adrenergic receptor subtype(s) in different vascular beds.

Activation of the sympathetic nervous system and catecholamine release is a well-known consequence of OSA and levels of catecholamines correlate with the cardiovascular end points, such as hypertension (Elmasry et al., 2002; Bisogni et al., 2016). Human studies have shown that experimental IH increases sympathetic nervous system activation and release of catecholamines (Foster et al., 2009). Our studies confirm that IH causes the release of catecholamines and consequently increases the levels of epinephrine. Importantly, we found that IH-induced catecholamines, specifically epinephrine, are required for the EC activation.

One key finding of our study is the observed differences in the response of ECs to IH between *in vitro* and *in vivo* models. Although we used established models of IH, observed differences may have been affected by the differences in the duration of IH between the two models. Moreover, our assessment of the effect of IH on ECs was limited to cytokines and adhesion molecules. Further studies using unbiased approaches, such as RNA-seq, will be needed to provide a better understanding of the effect of IH and catecholamines on EC activation. Due to the small number of cells, we used all ECs isolated from the whole aorta to assess gene expression responses to IH *in vivo.* A recent single-cell RNA-seq study reported heterogeneity in EC populations within and between tissues (Feng et al., 2019) as well as sex- and age-related differences (Huang et al., 2021). Feng et al. identified three different populations of ECs in aorta based on uniquely expressed genes (Feng et al., 2019). We have also previously reported differences in gene expression in aortic ECs based on the location and consequently exposure to different types of blood flow (Wu et al., 2017). Further studies will help to determine the effects of IH on the subpopulations of ECs.

While it is not possible to perfectly mimic OSA by exposing mice or cells to IH exposure, our published data, measuring blood gases, and other reference points support the use of *in vitro* and *in vivo* models of IH that we employed in our studies (Peng and Prabhakar, 2003, 2004). We did not measure blood pressure in the current study as an endpoint to determine whether mice were exposed equally to IH; however, we have recently reported blood pressure responses to IH in non-sedated mice using tail-cuff method as well as a telemetry approach (Peng et al., 2006, 2021). Both approaches showed significant elevation of both systolic and diastolic blood pressure in C57BL/6 mice treated with IH for 10 days. Importantly, there was low variability in the blood pressure response following IH.

OSA is an independent risk factor for CVD (Young et al., 1993, 2009; Somers et al., 1995; Fletcher, 2001; Neubauer, 2001; Marin et al., 2005; Yaggi et al., 2005; Marshall et al., 2008; Punjabi, 2008; Dempsey et al., 2010; AASM, 2014). The frequency of CVD increases with the severity of OSA (Marin et al., 2005), and some of the cardiovascular effects of OSA, such as hypertension, show linear association with the severity of hypoxia (McNicholas et al., 2007). Our findings provide another important mechanism by which IH can contribute mechanistically to the development of CVD through induction of EC activation. These findings suggest that IH-induced sympathetic nervous system activation and catecholamines can be targeted to prevent the cardiovascular consequences of OSA.

## Data Availability Statement

The raw data supporting the conclusions of this article will be made available by the authors, without undue reservation.

## Ethics Statement

The animal study was reviewed and approved by the University of Chicago IACUC.

## Author Contributions

RC-A, YF, RH, NP, and GM conceived the idea and designed the experiments. RC-A, AM, DW, PW, KS, Y-JP, JN, XS, and RH performed the experiments and collected the data. RC-A, AM, DW, PW, KS, XS, RH, NP, and GM performed the data analysis and interpretation. RC-A, AM, YF, RH, NP, and GM drafted and edited the manuscript. Final approval of manuscript was done by all authors.

### Conflict of Interest

The authors declare that the research was conducted in the absence of any commercial or financial relationships that could be construed as a potential conflict of interest.
