# Supplementary material for: Intermittent Hypoxia-Induced Activation of Endothelial Cells Is Mediated via Sympathetic Activation-Dependent Catecholamine Release
Source: Front Physiol. 2021 Jul 12;12:701995. doi: 10.3389/fphys.2021.701995 (PMC8311436; doi:10.3389/fphys.2021.701995)
Supplement: Supplementary file 1 [file Data_Sheet_1.PDF]

SUPPLEMENTARY INFORMATION

Intermittent hypoxia-induced activation of endothelial cells is mediated via sympathetic activation dependent catecholamine release

Rengul Cetin-Atalay[1,2]*, Angelo Y. Meliton[1,2]*, David Wu[1,2], Parker S. Woods[1,2], Kaitlyn A. Sun[1,2], Ying-Jie Peng[1,3,4], Jayasri Nanduri[1,3,4], Yun Fang[1,2,4], Robert B. Hamanaka[1,2,4], Nanduri Prabhakar[1,3,4], and Gökhan M. Mutlu[1,2,4]

[1]Department of Medicine, [2]Section of Pulmonary and Critical Care Medicine, and [3]Section of Emergency Medicine and [4]Institute for Integrative Physiology, University of Chicago, Chicago, Illinois, United State

Supplementary Figure 1

Normoxia IH

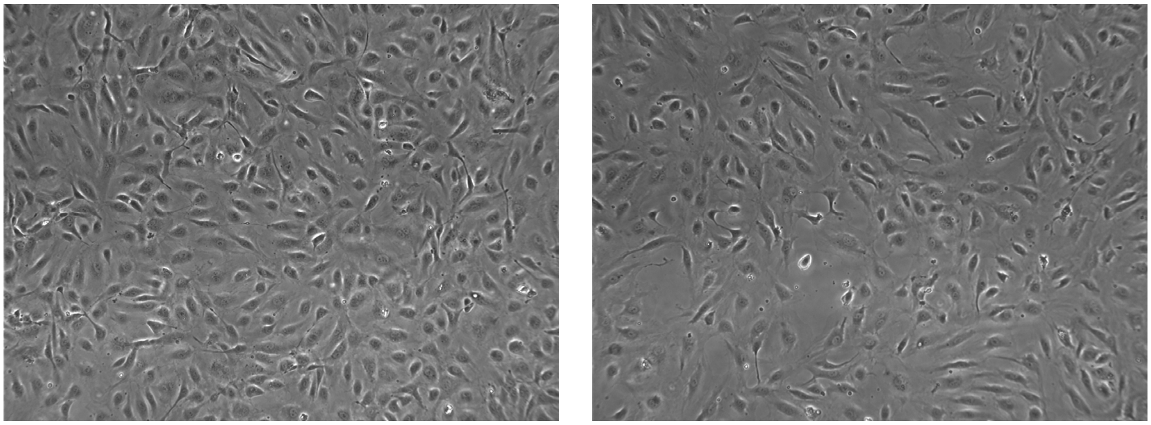

Supplementary Figure 1. IH does not affect the morphology of endothelial cells. Images of endothelial cells were taken after 60 cycles of IH compared to control normoxia-exposed cells.

Supplementary Figure 2

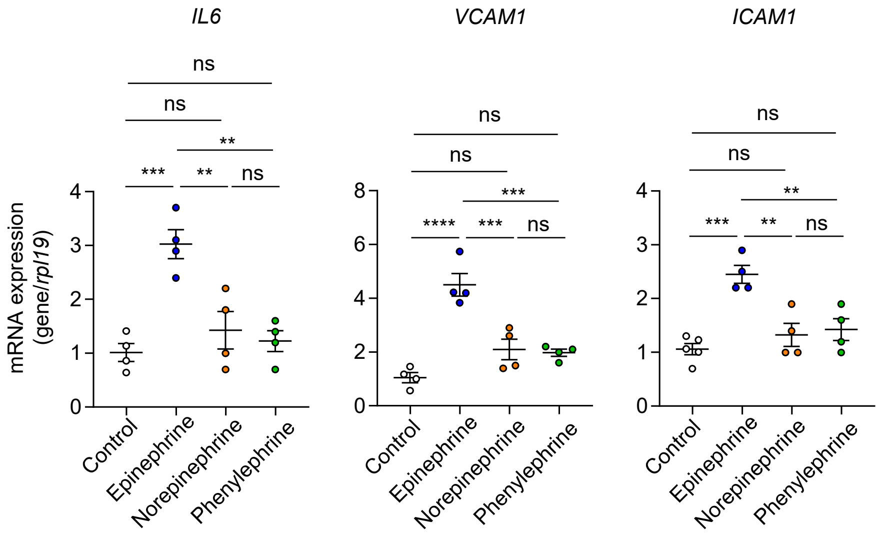

Supplementary Figure 2. Epinephrine but not norepinephrine or phenylephrine induces EC activation *in vitro*. We treated ECs under normoxia with epinephrine (10μM), norepinephrine (10μM), and phenylephrine (10μM) for 24 hours and then assessed EC activation by measuring mRNA expression of markers of EC activation genes including *IL6*, *VCAM1* and *ICAM1* (N=4). ns= not significant. **p<0.005, ***p<0.001, ****p<0.0001.
